# Supplementary material for: Uncovering new families and folds in the natural protein universe
Source: Nature. 2023 Sep 13;622(7983):646–53. doi: 10.1038/s41586-023-06622-3 (PMC10584680; doi:10.1038/s41586-023-06622-3)
Supplement: Supplementary file 1 — Reporting Summary [file 41586_2023_6622_MOESM1_ESM.pdf]

nature portfolio

Corresponding author(s): Joana Pereira
Torsten Schwede

Last updated by author(s): 29.08.2023

Reporting Summary

Nature Portfolio wishes to improve the reproducibility of the work that we publish. This form provides structure for consistency and transparency in reporting. For further information on Nature Portfolio policies, see our Editorial Policies and the Editorial Policy Checklist.

Statistics

For all statistical analyses, confirm that the following items are present in the figure legend, table legend, main text, or Methods section.

n/a	Confirmed	
☐	☒	The exact sample size (*n*) for each experimental group/condition, given as a discrete number and unit of measurement
☐	☒	A statement on whether measurements were taken from distinct samples or whether the same sample was measured repeatedly
☐	☒	The statistical test(s) used AND whether they are one- or two-sided<br>*Only common tests should be described solely by name; describe more complex techniques in the Methods section.*
☒	☐	A description of all covariates tested
☒	☐	A description of any assumptions or corrections, such as tests of normality and adjustment for multiple comparisons
☐	☒	A full description of the statistical parameters including central tendency (e.g. means) or other basic estimates (e.g. regression coefficient) AND variation (e.g. standard deviation) or associated estimates of uncertainty (e.g. confidence intervals)
☐	☒	For null hypothesis testing, the test statistic (e.g. *F*, *t*, *r*) with confidence intervals, effect sizes, degrees of freedom and *P* value noted<br>*Give P values as exact values whenever suitable.*
☒	☐	For Bayesian analysis, information on the choice of priors and Markov chain Monte Carlo settings
☒	☐	For hierarchical and complex designs, identification of the appropriate level for tests and full reporting of outcomes
☐	☒	Estimates of effect sizes (e.g. Cohen's *d*, Pearson's *r*), indicating how they were calculated

Our web collection on statistics for biologists contains articles on many of the points above.

Software and code

Policy information about availability of computer code

Data collection	Data collection for the annotated network was carried out using custom code available at https://github.com/ProteinUniverseAtlas/dbuilder which uses Python 3.6 and PyMongo v3.11.3.<br>Training data for protein substructure decomposition and outlier detection was created using custom code available at https://github.com/TurtleTools/geometricus/tree/master/training using Python 3.9, cath-tools-genomescan (version 17/12/2019), and ProteinNet (CASP12 dataset)
Data analysis	Custom code for data analysis can be found at https://github.com/ProteinUniverseAtlas/AFDB90v4 and uses:<br>Python 3.6, 3.9<br>SciPy (v1.5.4)<br>NetworkX (v2.5.1)<br>ProDy (v2.2.0)<br>Geometricus (v0.5.0)<br>PyTorch (v1.12.0)<br>Gensim (v4.2.0)<br>scikit-learn (v1.1.1)<br>Datashader (v0.12.1)<br><br>In addition, the following tools were used for analyses as described in the Methods:<br>MMseqs (release 13-45111)<br>MUSCLE (v5.1)<br>GCsnap (v1.0.17)

DeepFRI (v1.0.0)
Foldseek (Version: 7.04e0ec8)
AlphaFold (v2.3.0)
PyMol (open-source v2.5.0)

For manuscripts utilizing custom algorithms or software that are central to the research but not yet described in published literature, software must be made available to editors and reviewers. We strongly encourage code deposition in a community repository (e.g. GitHub). See the Nature Portfolio guidelines for submitting code & software for further information.

Data

Policy information about availability of data

All manuscripts must include a data availability statement. This statement should provide the following information, where applicable:

- Accession codes, unique identifiers, or web links for publicly available datasets
- A description of any restrictions on data availability
- For clinical datasets or third party data, please ensure that the statement adheres to our policy

All data used for this study is publicly available in UniProtKB (https://www.uniprot.org/, UniRef version 2022_03), the AlphaFold database (https://alphafold.ebi.ac.uk/, version 4, with specific examples corresponding to UniProt IDs A0A0E3S9F7, A0A3R7AQ40, A0A520JWH3, A0A1W9UY89, A0A7J4P9B0, A0A0F9A5W1, A0A0P9GTS8, A0A418VYX3, A0A2S5M855, A0A2K2VML8, A0A098EYBO, G0TGH8, A0A015IZK3, A0A377W562, A0A494VZL1, A0A0S7BXY3, A0A7X7MB17, YFHO_BACSU, A8JBY2_CHLRE, and A0A3A8FAL8), the CATH database (https://www.cathdb.info/, version 4.2.0), ProteinNet (https://github.com/aqlaboratory/proteinnet, CASP12 dataset), Foldseek benchmark data (https://wwwuser.gwdg.de/~compbiol/foldseek), the Protein Data Bank (https://www.ebi.ac.uk/pdbe/, PDB IDs 5FMT, 5GKH, 8D3P, 6SK0, 2FIM, 1ZXU, 6GXC and 7OCI), and NCBI GenBank (https://www.ncbi.nlm.nih.gov/protein/, EntrezIDs WP_213381069.1 and WP_213381068.1).
For the laboratory experiments all data generated are included in the manuscript and supplementary materials. All data and metadata generated supporting the large and the individual sequence similarity networks are available at https://zenodo.org/record/8121336 (CC-BY 4.0). An interactive version of the large sequence similarity network, queryable by keyword, UniProt ID, connected component ID, community ID, protein sequence, and protein structure, is available at https://uniprot3d.org/atlas/AFDB90v4. The interactive resource allows also for the downloading of the metadata associated with each individual connected component and community, as well as for the results of any search.

Research involving human participants, their data, or biological material

Policy information about studies with human participants or human data. See also policy information about sex, gender (identity/presentation), and sexual orientation and race, ethnicity and racism.

Reporting on sex and gender	Not applicable. No human participants or human data was used in this study.
Reporting on race, ethnicity, or other socially relevant groupings	Not applicable. No human participants or human data was used in this study.
Population characteristics	Not applicable. No human participants or human data was used in this study.
Recruitment	Not applicable. No human participants or human data was used in this study.
Ethics oversight	Not applicable. No human participants or human data was used in this study.

Note that full information on the approval of the study protocol must also be provided in the manuscript.

Field-specific reporting

Please select the one below that is the best fit for your research. If you are not sure, read the appropriate sections before making your selection.

☒ Life sciences ☐ Behavioural & social sciences ☐ Ecological, evolutionary & environmental sciences

For a reference copy of the document with all sections, see nature.com/documents/nr-reporting-summary-flat.pdf

Life sciences study design

All studies must disclose on these points even when the disclosure is negative.

Sample size	We followed the standard practices in the toxin-antitoxin molecular microbiology field. The sample size of three is regularly used in toxin-antitoxin microbiology field for growth and metabolic labeling experiments. The effects were strong and do not require further statistical analysis.
Data exclusions	No data were excluded.
Replication	The experiments were repeated in at least three biological independent replicates. All of the attempts were successful and showed the same results.

Randomization	We followed the standard practices in the toxin-antitoxin molecular microbiology field. Randomization of samples is generally not practiced.
Blinding	We followed the standard practices in the toxin-antitoxin molecular microbiology field. Blinding is generally not practiced.

Reporting for specific materials, systems and methods

We require information from authors about some types of materials, experimental systems and methods used in many studies. Here, indicate whether each material, system or method listed is relevant to your study. If you are not sure if a list item applies to your research, read the appropriate section before selecting a response.

Materials & experimental systems

n/a	Involved in the study	
☒	☐	Antibodies
☒	☐	Eukaryotic cell lines
☒	☐	Palaeontology and archaeology
☒	☐	Animals and other organisms
☒	☐	Clinical data
☒	☐	Dual use research of concern
☒	☐	Plants

Methods

n/a	Involved in the study	
☒	☐	ChIP-seq
☒	☐	Flow cytometry
☒	☐	MRI-based neuroimaging
